# Preclinical evaluation of carbon-11 and fluorine-18 sulfonamide derivatives for *in vivo* radiolabeling of erythrocytes

**DOI:** 10.1186/2191-219X-3-4

**Published:** 2013-01-15

**Authors:** Olivier Gheysens, Vamsidhar Akurathi, Rufael Chekol, Tom Dresselaers, Sofie Celen, Michel Koole, Dieter Dauwe, Bernard J Cleynhens, Piet Claus, Stefan Janssens, Alfons M Verbruggen, Johan Nuyts, Uwe Himmelreich, Guy M Bormans

**Affiliations:** 1 Nuclear Medicine, University Hospital Leuven, Herestraat 49, Leuven BE-3000, Belgium; 2 Department of Imaging and Pathology, Katholieke Universiteit Leuven, Herestraat 49, Leuven, BE-3000, Belgium; 3 Laboratory of Radiopharmacy, Katholieke Universiteit Leuven, O&N2, Herestraat 49, Box 821, Leuven, BE-3000, Belgium; 4 Biomedical NMR Unit, Katholieke Universiteit Leuven, O&N2, Herestraat 49, Leuven, BE-3000, Belgium; 5 Cardiovascular Diseases, University Hospital Leuven, Herestraat 49, Leuven, BE-3000, Belgium; 6 Division of Cardiology, Department of Cardiovascular Sciences, Katholieke Universiteit Leuven, Herestraat 49, Leuven, BE-3000, Belgium; 7 Division of Imaging and Cardiovascular Dynamics, Department of Cardiovascular Sciences, Katholieke Universiteit Leuven, Herestraat 49, Leuven, BE-3000, Belgium

**Keywords:** Blood pool imaging, Carbonic anhydrases, PET tracers, Sulfonamides

## Abstract

**Background:**

To date, few PET tracers for *in vivo* labeling of red blood cells (RBCs) are available. In this study, we report the radiosynthesis and *in vitro* and *in vivo* evaluation of ^11^C and ^18^F sulfonamide derivatives targeting carbonic anhydrase II (CA II), a metallo-enzyme expressed in RBCs, as potential blood pool tracers. A proof-of-concept *in vivo* imaging study was performed to demonstrate the feasibility to assess cardiac function and volumes using electrocardiogram (ECG)-gated positron emission tomography (PET) acquisition in comparison with cine magnetic resonance imaging (cMRI) in rats and a pig model of myocardial infarction.

**Methods:**

The inhibition constants (*K*_i_) of CA II were determined *in vitro* for the different compounds by assaying CA-catalyzed CO_2_ hydration activity. Binding to human RBCs was estimated after *in vitro* incubation of the compounds with whole blood. Biodistribution studies were performed to evaluate tracer kinetics in NMRI mice. ECG-gated PET acquisition was performed in Wistar rats at rest and during pharmacological stress by infusing dobutamine at 10 μg/kg/min and in a pig model of myocardial infarction. Left ventricular ejection fraction (LVEF) and volumes were compared with values from cMRI.

**Results:**

The *K*_i_ of the investigated compounds for human CA II was found to be in the range of 8 to 422 nM. The fraction of radioactivity associated with RBCs was found to be ≥90% at 10- and 60-min incubation of tracers with heparinized human blood at room temperature for all tracers studied. Biodistribution studies in mice indicated that 30% to 67% of the injected dose was retained in the blood pool at 60 min post injection. A rapid and sustained tracer uptake in the heart region with an average standardized uptake value of 2.5 was observed from micro-PET images. The LVEF values obtained after pharmacological stress in rats closely matched between the cMRI and micro-PET values, whereas at rest, a larger variation between LVEF values obtained by both techniques was observed. In the pig model, a good agreement was observed between PET and MRI for quantification of left ventricular volumes and ejection fraction.

**Conclusions:**

The ^11^C and ^18^F sulfonamide derivatives can be used for efficient *in vivo* radiolabeling of RBCs, and proof-of-concept *in vivo* imaging studies have shown the feasibility and potential of these novel tracers to assess cardiac function.

## Background

Radiolabeled red blood cells (RBCs) are used in the assessment of blood pool dynamics, for investigating angiofibroma and gastrointestinal hemorrhage and for localizing intramuscular hemangioma [1-5]. To date, very few positron emission tomography (PET) tracers have been reported as RBC labeling agents. ^68^Ga-oxine has been used for the labeling of RBCs, but this requires an *in vitro* labeling procedure [6]. Examples of agents for *in vivo* RBC labeling are ^11^CO and C^15^O in gaseous form [7-9]. Due to *in vivo* instability and as a requirement of the sophisticated equipment for inhalation of these tracer gases, extensive usage of these tracers in preclinical or clinical studies is limited. Recently, human serum albumin (HSA) and rat serum albumin (RSA) were labeled with ^62^Cu and ^68^Ga, but one of the limitations with ^62^Cu is the *in vivo* instability of the complex, and ^68^Ga-DOTA-HSA has yet to prove its clinical application [10-12].

Radiolabeling of rat and human RBCs by specific enzyme-inhibitor approach was first reported in 1991 by Singh et al. with ^123^I- or ^125^I-labeled *p-*iodobenzenesulfonamide targeting carbonic anhydrase I and II (CA I and II), a metalloenzyme found in RBCs [13,14]. The authors found that the activity associated with RBCs decreased to about 50% of the injected dose (ID) at 24 h post injection (p.i.) in rats, whereas more than 88% of the ID remained associated with RBCs at 24 h p.i. in human volunteers. Fluorine-18 has appealing characteristics as a radionuclide for PET as large batches (>500 GBq) of fluorine-18 can be produced by current cyclotrons, and the half-life of 110 min allows distribution from the production site to multiple remote PET sites.

The short half-life of carbon-11 precludes its distribution but results in lower radiation burden to the patient and allows the combination of scans with different PET tracers in a 1-day protocol.

In this study, we report the evaluation of ^11^C- and ^18^F-labeled sulfonamides for *in vitro* and *in vivo* labeling of RBCs. In addition, a proof-of-concept *in vivo* imaging study was carried out with 4-(2-[^18^F]fluoroethoxy)benzenesulfonamide, [^18^F]-(**5**), to assess the feasibility to calculate left ventricular (LV) volumes and ejection fraction (EF) in comparison with cine magnetic resonance imaging (cMRI). Currently, LV function and volumes are most commonly assessed by conventional equilibrium-gated radionuclide angiography and echocardiography. The latter is a widely available technique with easy access but is limited by a high inter- and intra-observer variability and compromised echogenicity in obese patients. More recently, the cMRI technique is more widely used in clinical routine to estimate left ventricular ejection fraction (LVEF) values and heart function, especially in patients with dilated or distorted ventricles, due to its high-resolution functional images. Echocardiography and MRI are based on several assumptions of LV geometry which may not hold true in dysfunctional LV in contrast with radionuclide-based techniques that are less dependent on geometrical assumptions. In addition, for patients with pacemakers, defibrillators, or other implanted electronic devices that preclude cMRI, PET imaging offers a valuable alternative for cMRI. Therefore, PET blood pool agents that have a potential to assess cardiac function are an added value in nuclear cardiology.

## Methods

### Chemistry and radiochemistry

In detail description of synthesis of precursors, reference analogs (**1** to **5**) and production of the secondary radiolabeling agents ^11^CH_3_I and ^18^FEtBr are summarized in Additional file 1.

#### 4-(2-[^18^F]fluoroethoxy)benzoyl aminoethylbenzenesulfonamide [^18^F]-(3) and 4-[^11^C]methoxybenzoyl aminoethylbenzenesulfonamide [^11^C]-(4)

The synthons ^11^CH_3_I or ^18^FEtBr were bubbled into a solution consisting of the phenolic precursor, 4-hydroxy-*N*-[2-(4-sulfamoylphenyl)ethyl]benzamide (**2**, 0.2 mg) and Cs_2_CO_3_ (1 to 2 mg) in anhydrous dimethylformamide (DMF). The reaction mixture was heated at 90°C for 5 min (^11^CH_3_I) or 15 min (^18^FEtBr). The reaction mixture was diluted with water (1 mL) and injected onto a high-performance liquid chromatography (HPLC) system (XBridge C_18_ column, 5 μm, 4.6 × 150 mm; Waters Corporation, Milford, MA, USA) eluted with a mixture of 0.05 M NaOAc (pH 5.5) and EtOH (80:20 *v*/*v*) at a flow rate of 1 mL/min. UV detection of the HPLC eluate was performed at 254 nm. The radiolabeled product [^18^F]**-**(**3**) was collected after 15 min and [^11^C]**-**(**4**) eluted 11 min after injection on the HPLC system. The collected peak corresponding to the desired radioligand was then diluted with saline (Mini Plasco®, Braun, Melsungen, Germany) to obtain a final EtOH concentration of ≤5%, and the solution was filtered through a sterile 0.22-μm membrane filter (Millex®-GV, Millipore Co., Billerica, MA, USA). Quality control was performed on an analytical HPLC system consisting of an XBridge C_18_ column (3.5 μm, 3 × 100 mm; Waters Corporation) eluted with a mixture of 0.05 M NaOAc buffer (pH 5.5) and acetonitrile (80:20 *v*/*v*) at a flow rate of 0.8 mL/min. UV detection was performed at 254 nm. The tracers [^18^F]**-**(**3**) and [^11^C]**-**(**4**) were eluted at 10 and 5 min, respectively, and their identity was confirmed by co-elution with authentic nonradioactive reference solutions. The tracers [^18^F]**-**(**3**) and [^11^C]**-**(**4**) were synthesized with a decay-corrected radiochemical yield of 45% and 30% (*n* = 3), respectively (relative to the starting radioactivity of ^18^FEtBr and ^11^CH_3_I), and with a radiochemical purity of ≥98%. Starting from ^18^FEtBr and ^11^CH_3_I, the synthesis time to obtain the pure product was 50 ± 10 min for [^18^F]**-**(**3**) and 40 ± 5 min for [^11^C]**-**(**4**). The average specific activity was found to be in the range of 37 to 71 GBq/μmol at the end of synthesis (EOS).

#### 4-(2-[^18^F]fluoroethoxy)benzenesulfonamide [^18^F]-(5) and 4-[^11^C]methoxybenzene sulfonamide [^11^C]-(6)

The synthons ^18^FEtBr or ^11^CH_3_I were bubbled into a solution of the phenolic precursor 4-hydroxybenzene-1-sulfonamide (0.8 mg) in a mixture of 1 M NaOH (2.5 μL) and DMF (0.3 mL). The mixture was heated at 90°C for 5 min (^11^CH_3_I) or 15 min (^18^FEtBr). The crude mixture was diluted with water (1 mL) and injected onto an HPLC column (XTerra C_18_, 5 μm, 7.8 × 150 mm; Waters Corporation) eluted with a mixture of 0.05 M NH_4_OAc (pH 6.8) and EtOH (90:10 *v*/*v*) at a flow rate of 2 mL/min. UV detection of the HPLC eluate was performed at 254 nm. The radiolabeled product [^18^F]**-**(**5**) was collected after 16 min, and [^11^C]**-**(**6**) was eluted after 15 min on the HPLC system. The collected peak corresponding to the desired radioligand was then diluted with saline (Mini Plasco®, Braun, Melsungen, Germany) to obtain a final EtOH concentration of ≤5%, and the solution was sterile filtered through a 0.22-μm membrane filter (Millex®-GV, Millipore Co.). QC was performed on an analytical HPLC system consisting of an XTerra C_18_ column (5 μm, 4.6 × 250 mm; Waters Corporation). For [^18^F]**-**(**5**), the mobile phase was a mixture of 0.05 M NH_4_OAc (pH 6.8) and EtOH (80:20 *v*/*v*); for [^11^C]**-**(**6**), a mixture of 0.05 M NH_4_OAc (pH 6.8) and acetonitrile (80:20 *v*/*v*) was used. The flow rate was 0.9 mL/min. UV detection was performed at 254 nm. The tracers [^18^F]**-**(**5**) and [^11^C]**-**(**6**) were eluted at 11 and 9 min, respectively, and the identity of the tracers was confirmed by co-elution with authentic nonradioactive reference solutions. The tracers [^18^F]**-**(**5**) and [^11^C]**-**(**6**) were synthesized with a decay-corrected radiochemical yield of 65% (*n* = 3) and with a radiochemical purity of ≥98%. Starting from ^18^FEtBr and ^11^CH_3_I, the synthesis time to obtain the pure product was 55 ± 10 min for [^18^F]**-**(**5**) and 50 ± 5 min for [^11^C]**-**(**6**). The average specific activity for both tracers was found to be 90 GBq/μmol at the EOS.

### *In vitro* studies

#### Log D (1-octanol/phosphate buffer pH 7.4)

Determination of the distribution coefficient (log D_1-octanol/phosphate buffer pH 7.4_), was carried out by a shake flask method [15]. An aliquot (25 μL) of the tracer agents ^18^F]**-**(**3**), ^11^C]**-**(**4**), ^18^F]**-**(**5**), or ^11^C]**-**(**6**) (185 to 555 kBq/mL) was added to a polypropylene tube (5 mL; Sarstedt, Nümbrecht, Germany) containing 2 mL of 0.025 M sodium phosphate buffer pH 7.4 and 2 mL of 1-octanol. The tube was vortexed for 2 min at room temperature followed by centrifugation at 3,000 rpm for 10 min (Eppendorf centrifuge 5810, Eppendorf, Westbury, NY, USA). Aliquots of 50 μL of the 1-octanol phase and 500 μL of the phosphate buffer phase were pipetted into separate tared Eppendorf tubes with adequate care to avoid cross contamination between the two phases. The samples were weighed, and radioactivity was quantified using an automated gamma counter. The experiments were carried out sixfold*.*

#### Determination of inhibition constant

The inhibition constants (*K*_i_) of the reference analogs (**3**), (**4**), (**5**), and (**6**) against human CA (hCA) I and II isozymes were determined by assaying the CA-catalyzed CO_2_ hydration activity [16], using Applied Photophysics' (Leatherhead, UK) stopped-flow instrument. Phenol red (0.2 mM) was used as indicator, working at the absorbance maximum of 557 nm, with 10 mM Hepes as buffer (pH 7.5) and 0.1 M Na_2_SO_4_ (for maintaining constant ionic strength) at 25°C following the CA-catalyzed CO_2_ hydration reaction for a period of 10 to 100 s (the uncatalyzed reaction needs around 60 to 100 s under assay conditions, whereas the catalyzed reactions take around 6 to 10 s). The CO_2_ concentrations ranged from 1.7 to 17 mM for the determination of kinetic parameters. Each compound was tested in the concentration range between 0.01 nM to 100 μM. The uncatalyzed rates were determined in the same manner and subtracted from the total observed rates. Stock solutions of the compounds (0.1 mM) were prepared in distilled water with 10% to 20% (*v*/*v*) DMSO (which is non-inhibitory at these concentrations), and dilutions up to 0.01 nM were made with distilled water. Inhibitor and enzyme solutions were preincubated together for 15 min at room temperature prior to the assay, in order to allow the formation of enzyme-inhibitor complex. The inhibition constants were obtained by nonlinear least square methods using PRISM 3 (GraphPad Software, La Jolla, CA, USA), and they represent the mean from at least three different determinations.

#### Whole blood analysis

Blood samples from a healthy human volunteer were collected in a BD vacutainer™ (4.5 mL; containing lithium heparin; BD, Franklin Lakes, NJ, USA). Aliquots of whole blood (0.4 mL) were incubated with [^11^C]**-**(**4**) or [^11^C]**-**(**6**) (370 kBq/0.1 mL) for 10 min and with [^18^F]**-**(**3**) or [^18^F]**-**(**5**) (37 kBq/0.1 mL) for 60 min at room temperature. A different incubation time with ^11^C and ^18^F tracer agents was used considering the radionuclides' half-life (^11^C, 20 min; ^18^F, 110 min). After incubation for 10 or 60 min, the plasma was separated from the blood cells by centrifugation at 3,000 rpm (1,837×*g*) for 5 min (Eppendorf centrifuge 5810). To remove residual plasma and unbound tracer from the RBCs, phosphate-buffered saline (PBS) pH 7.4 (0.4 mL) was added to the cell fraction. After incubation for 2 min, the PBS was separated from the RBCs using centrifugation. This rinsing procedure was carried out twice. For competition studies with acetazolamide (AAZ), the same procedure was followed as mentioned afore. To the whole blood and tracer agent mixture, a solution of AAZ (0.1 mL) was added to result final concentrations of 0.01, 0.1, 0.2, 0.3, 0.4, 0.5, and 1.0 mM, and as a control, PBS (0.1 mL, PBS pH 7.4) was added. The radioactivity associated with the RBCs and plasma was quantified using an automated gamma counter.

#### Distribution of activity within the blood

Human blood (12 mL) was collected in a syringe containing 2.4 mL ACD (citrate-dextrose solution), and 3.7 MBq was added and gently mixed. Two milliliters of a 2% methylcellulose was added, gently mixed, and allowed to stand for 60 min so that the RBCs sediment by gravity. The supernatant was carefully drawn and centrifuged at 1,000 rpm (140×*g*) for 5 min. The plasma-rich supernatant and white blood cell (WBC)-rich pellet were separated [17]. The activity in the three fractions was counted using an automated gamma counter. The procedure was carried out in triplicate.

### *In vivo* studies

#### Biodistribution studies

The biodistribution studies were performed in wild-type NMRI mice with body weights ranging from 30 to 40 g. Mice were intravenously injected with 0.1 MBq of ^18^F]**-**(**3**) or ^18^F]**-**(**5**) and 5.5 MBq of ^11^C]**-**(**4**) or ^11^C]**-**(**6**) under anesthesia (2% isoflurane in O_2_ at a flow rate of 1 L/min). The animals were killed by decapitation at 2 min or 60 min p.i. (*n* = 4/time point for ^18^F]**-**(**3**), ^11^C]**-**(**4**), and ^11^C]**-**(**6**); *n* = 6 for ^18^F]**-**(**5**)). Blood was collected, and all major organs were dissected and collected in tarred tubes and were weighed. The radioactivity in each organ was counted using an automated gamma counter, corrected for background radioactivity and expressed as follows: percentage of the injected dose (% ID) or as standardized uptake value (SUV) (SUV = (Counts in tissue per gram of tissue) / (Injected counts per total body mass (g)). For the calculation of total radioactivity in the blood, blood mass was assumed to be 7% of the body mass [18].

#### Small animal imaging studies

PET images were acquired on a FOCUS 220 tomograph (Siemens/Concorde Microsystems, Knoxville, TN, USA). During all scan sessions, rats were anesthetized (2.5% isoflurane in O_2_ at a flow rate of 1 L/min) and scanned in prone position. [^18^F]**-**(**5**) (37 MBq) was administered via the tail vein, and a 1-h dynamic scan was acquired. The images were acquired in list mode and binned in sinograms using a 21-dynamic frame protocol (4 × 15, 4 × 60, 5 × 180, 8 × 300 s). Reconstruction was performed with the Focus 220 software (using Fourier rebinning, followed by two-dimensional (2D) OSEM algorithm), and data were analyzed using PMOD (version 2.65; PMOD, Zurich, Switzerland). The radioactivity concentration in the heart region was expressed as SUVs as a function of time after injection of the radiotracer.

An electrocardiogram (ECG)-gated micro-PET scan was performed with anesthetized (2.5% isoflurane in O_2_ at a flow rate of 1 L/min) Wistar rats (*n* = 2) after administration of 48 MBq/0.7 mL of [^18^F]**-**(**5**) via the tail vein. The acquisition was carried out at rest (0.5 h) and stress (0.5 h) by infusion of dobutamine at 10 μg/kg/min. The acquired data were reconstructed into a series of 12 ECG-gated images. To reduce noise, images were filtered with a 3D Gaussian filter (FWHM 1 mm) along the spatial dimensions and with a low pass filter (preserving only the mean and the first four harmonics) along the time dimension. A volume of interest containing the LV cavity in all 12 images was manually defined. For each image, the cavity volume was determined by applying a threshold of 50% of the maximum value inside that volume of interest.

Anesthetized rats also underwent cMRI with a 9.4-T 20-cm bore horizontal magnet using a linear resonator for excitation combined with a 2 × 2-phased array coil for detection (BRUKER Biospin, Ettlingen, Germany) and a retrospectively gated FLASH sequence (INTRAGATE®, BRUKER; repetition time (TR)/echo time (TE) = 7.6/1.8 ms, flip angle = 17°, matrix = 256 × 256, field of view (FOV) = 6 × 6 cm, 10 to 12 1-mm-thick short axis slices covering the LV, 15 frames reconstructed). To ensure stable and reproducible results, physiological parameters such as body temperature and respiratory and heart rates were carefully monitored throughout the imaging session, and it is noteworthy to mention that there was an interval of 7 to 10 days between the micro-PET and cMRI scan. A manual delineation of the endocardium, ignoring the papillary muscles, was carried out using a homemade software for cMRI [19]. The LVEF values were computed as follows: LVEF = (EDV − ESV/EDV) × 100, where EDV and ESV are end-diastolic volume and end-systolic volumes, respectively.

#### Pig study

Myocardial infarction was induced in 25- to 30-kg domestic pigs as previously described [20]. In brief, the left anterior descending coronary artery was temporarily occluded by a 90-min balloon inflation of a bare metal stent distal to the first diagonal branch. Continuous ECG and invasive pressure monitoring was registered during the whole procedure. Eight weeks later, gated blood pool PET and MRI were performed. ^18^F]**-**(**5**) (185 MBq) was administered via a venous catheter, and a 60-min ECG-gated PET scan was acquired in the list mode (HiRez Biograph 16, Siemens, Knoxville, TN, USA). A low-dose CT scan was conducted for attenuation correction. Based on the simultaneously recorded ECG signal, the cardiac cycle was divided in eight frames, and a PET sinogram was created from the list mode data for each frame. From these sinograms, eight images were reconstructed using 2D OSEM (five iterations and eight subsets) after Fourier rebinning. LV volumes and EF were determined in the same way as for the small animal study, i.e., by applying a threshold of 50% of the maximum in a manually defined volume of interest containing the cavity. The person who analyzed the PET data was blinded from the MRI results.

The MRI images were obtained in supine position on a 3-T unit (TRIO, Siemens, Erlangen, Germany) with ECG gating and during suspended respiration. A contiguous stack of short-axis images covering the entire ventricle was acquired with a 2D FLASH (fast low-angle shot) sequence using retrospective gating and the following imaging parameters: TR/TE = 25.45/2.39 ms, flip angle = 14°, matrix = 170 × 208, FOV = 310 × 380 mm, slice thickness = 6 mm, 40 cardiac phases, bandwidth 445 Hz/pixel). Volumes were determined by manual contouring in the same manner as described for the rat studies above.

All animal studies were approved by the Ethics Committee for Animal Experimentation (KU Leuven, Belgium) and were performed in accordance with the Guide for Care and Use of Laboratory Animals (NIH).

## Results and discussion

### Results

#### Chemistry and radiochemistry

**Figure 1:**
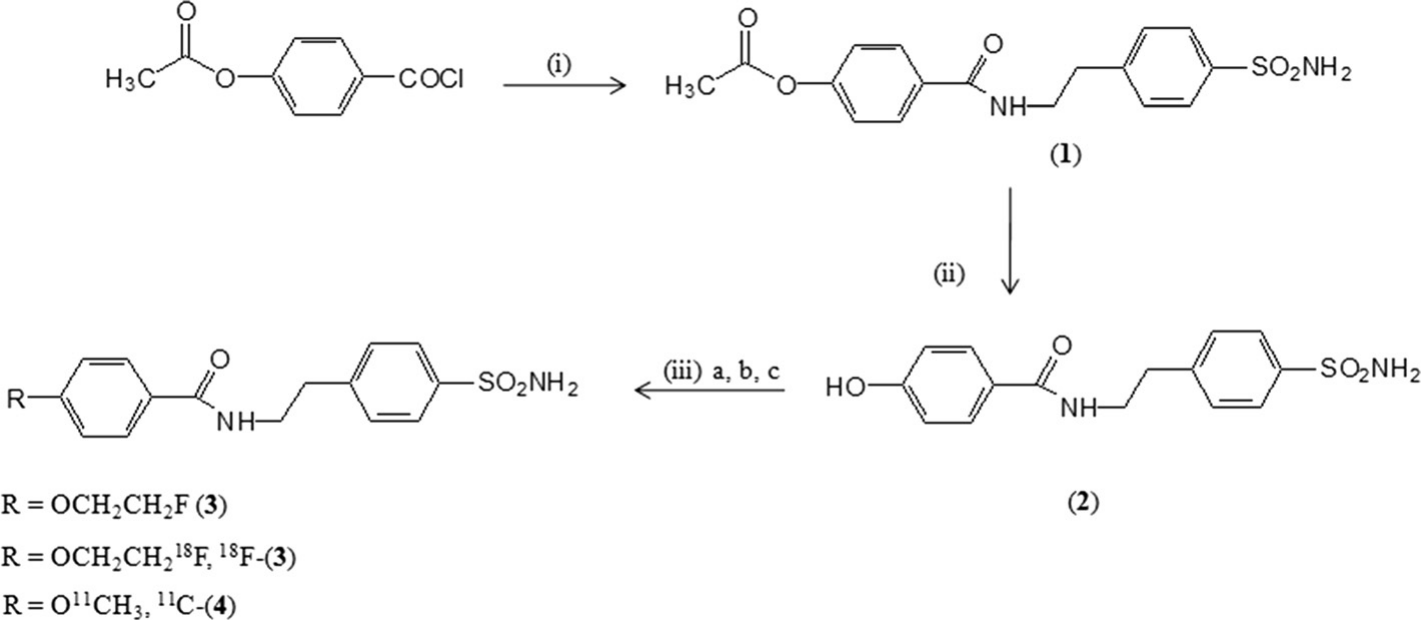
**Synthesis of [**^**18**^**F]-(3) and [**^**11**^**C]-(4). **Reagents and conditions: (i) AEBS, dichloromethane, pyridine for 72 h at room temperature, (ii) CH_3_ONa, HCL 1 M, and (iii) (a) DMF, 1-bromo-2-fluoroethane, 70°C, 16 h; (b) DMF, Cs_2_CO_3_, ^11^CH_3_I, 90°C, 15 min; (c) DMF, Cs_2_CO_3_, ^18^FEtBr, 90°C, 15 min.

**Figure 2:**
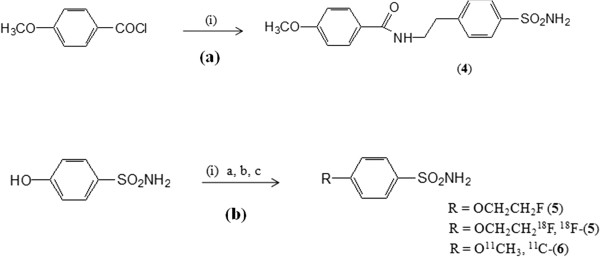
**Synthesis of [**^**18**^**F]-(5) and [**^**11**^**C]-(6).** Reagents and conditions: **(a)** (i) AEBS, dichloromethane, pyridine for 72 h at room temperature. **(b)** (i) **(a)** DMF, 1-bromo-2-fluoroethane, 70°C, 16 h; **(b)** DMF, NaOH 1 M, ^11^CH_3_I, 90°C, 15 min; (c) DMF, NaOH 1 M, ^18^FEtBr, 90°C, 15 min.

The *O*-acetyl protected intermediate (**1**) was obtained by treatment of 4-acetoxybenzoylchloride with 4-(2-aminoethyl-benzene)sulfonamide (AEBS) as described by Kuhnast et al. [21]. Hydrolysis of the acetyl protecting group resulted in the phenolic precursor (**2**) with a chemical yield of 80%. Alkylation of precursor (**2**) with 1-bromo-2-fluoro-ethane or 1-bromo-2-^18^F]fluoroethane (^18^FEtBr) resulted in the reference compound (**3**) and the radiolabeled fluoroethoxy derivative ^18^F]**-**(**3**), respectively. The ^11^C-labeled methoxyderivative ^11^C]**-**(**4**) was obtained similarly by alkylation of (**2**) with ^11^CH_3_I (Figure 1), and the reference compound (**4**) was synthesized by acylation of AEBS with 4-methoxybenzoyl chloride. Similarly, the other small (radiolabeled) sulfonamide derivatives, ^18^F]**-**(**5**) and ^11^C]**-**(**6**), were obtained by alkylation of commercially available 4-hydroxybenzene-1-sulfonamide (HBS) with ^18^FEtBr and ^11^CH_3_I, respectively. The reference compound of (**5**) has been synthesized similarly by alkylation of HBS with 1-bromo-2-fluoroethane (Figure 2). The authentic reference material (**6**) for ^11^C]**-**(**6**) was obtained commercially. All tracers were obtained with good radiochemical yield of 30% to 65% and high radiochemical purity (≥98%). The identity of the radiotracers was confirmed by co-elution with authentic reference compounds.

#### Log D_pH 7.4_, PSA, and K_i_

Table 1 summarizes log D, polar surface area (PSA), and the *in vitro K*_i_ data of reference compounds, along with AAZ.

**Table 1:** ***K***_**i **_**of reference compounds (3 to 6) against carbonic anhydrase isozymes hCA I and II**

**Compound**	***K***_**i **_**(nM)**^**a**^		**Log D**	**cPSA**^**d **^**(Å**^**2**^**)**
	**hCA I**^**b**^	**hCA II**^**b**^		
(**3**)	96	8	1.13	99
(**4**)	390	20	1.19	99
(**5**)	30,850	336	0.32	69
(**6**)	24,700	422	0.42	69
AAZ	250	12	−1.5^c^	109

For CO_2 _hydration reaction at 20°C. ^a^Standard errors of the mean in the range of 5% to 10% of the reported value (*n* = 3, different assays); ^b^human (cloned) isozymes; ^c^cLog D; ^d^cPSA = (calculated) polar surface area.

#### Whole blood analysis

**Figure 3:**
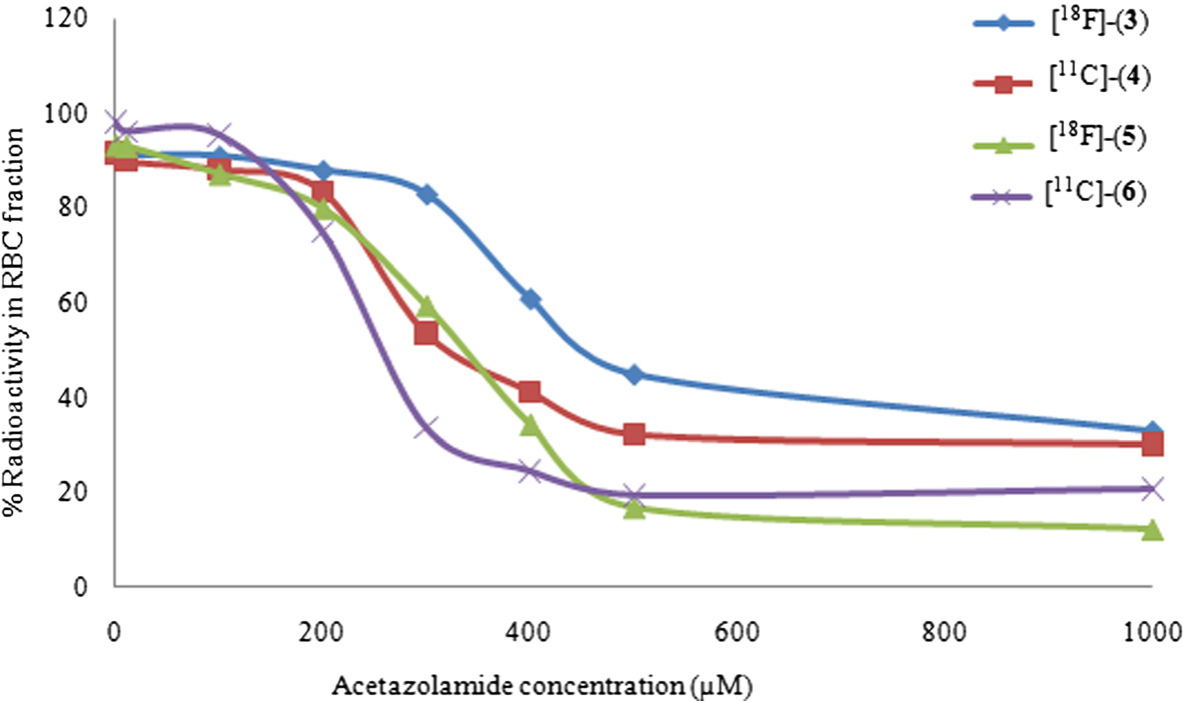
**Uptake of [**^**18**^**F]-(3), [**^**11**^**C]-(4), [**^**18**^**F]-(5), and [**^**11**^**C]-(6) in human RBCs versus AAZ concentration. **Data are expressed as percentage of radioactivity in the cell fractions.

The human whole blood analysis revealed that all four studied tracers had a rapid uptake, and ≥90% of the radioactivity was found in the RBCs after a 10- and 60-min incubation at RT. Furthermore, the radiotracer binding to carbonic anhydrases in RBCs was demonstrated from competitive inhibition studies by incubating the whole blood samples with radiotracers in the presence of the CA nonspecific inhibitor, AAZ, with final concentrations ranging from 10 μM to 1 mM. Figure 3 shows a sigmoid dose response curve with a reduction in the uptake of tracer (three- to eightfold) at concentrations ranging from 200 to 500 μM of AAZ, indicating a competitive binding of AAZ to CA I/II which is in agreement with an earlier report [22].

#### Distribution of activity within blood

The activity incubated with the blood was distributed in the RBC-rich sediment (92.5 ± 1.8%), and only 7.0 ± 1.6% and 0.5 ± 0.2% were found in the plasma-rich supernatant and WBC-rich compartment, respectively.

#### Biodistribution studies

Biodistribution studies in mice (Table 2) demonstrated a high blood pool retention of about ≥69% ID at 2 min p.i. and ≥52% ID at 60 min p.i. for all tracers, except in the case of [^18^F]**-**(**3**) where only 32% ID at 60 min p.i. was observed. All the other organs (the lungs, heart, brain, spleen, and pancreas) showed minimal tracer retention at 60 min p.i. The clearance of tracers [^18^F]**-**(**3**) and [^18^F]**-**(**5**) was mainly through the renal pathway (≥15% ID in urine at 60 min p.i.), whereas for [^11^C]**-**(**4**) and [^11^C]**-**(**6**), the clearance was mainly via the hepatobiliary pathway (12% to 19% ID in the intestines at 60 min p.i.). In the carcass, about 16% to 29% ID was observed at 2 and 60 min p.i. for all tracer agents; this is probably due to the activity associated with the residual blood in the carcass.

**Table 2:** **Biodistribution of [**^**18**^**F]-(3), [**^**11**^**C]-(4), [**^**18**^**F]-(5), and [**^**11**^**C]-(6) in normal NMRI mice**

**Organs**		**[**^**18**^**F]-(3)**		**[**^**11**^**C]-(4)**		**[**^**18**^**F]-(5)**		**[**^**11**^**C]-(6)**	
		**2 min**	**60 min**	**2 min**	**60 min**	**2 min**	**60 min**	**2 min**	**60 min**
Percentage of ID	Urine	0.2 ± 0.1	15.9 ± 6.0	0.4 ± 0.1	4.4 ± 2.7	0.3 ± 0.1	10.0 ± 5.0	0.3 ± 0.1	4.8 ± 0.3
	Kidneys	10.6 ± 0.8	2.8 ± 0.1	8.1 ± 1.3	4.1 ± 0.3	4.1 ± 0.8	3.7 ± 1.1	3.7 ± 0.1	4.1 ± 0.6
	Liver	15.8 ± 1.5	8.3 ± 1.2	8.4 ± 0.7	9.2 ± 1.1	6.0 ± 1.1	4.0 ± 0.2	6.9 ± 0.4	6.1 ± 0.5
	Spleen + pancreas	1.3 ± 0.2	0.9 ± 0.3	0.6 ± 0.1	0.8 ± 0.1	1.1 ± 0.1	0.9 ± 0.4	1.2 ± 0.1	2.3 ± 0.6
	Lungs	2.3 ± 0.5	0.7 ± 0.3	4.9 ± 2.5	4.2 ± 1.1	7.3 ± 3.9	1.8 ± 0.6	6.3 ± 2.7	3.1 ± 1.4
	Heart	0.8 ± 0.0	0.2 ± 0.2	0.5 ± 0.1	0.5 ± 0.1	0.6 ± 0.1	0.6 ± 0.2	0.7 ± 0.2	0.4 ± 0.1
	Intestines	5.9 ± 0.8	18.6 ± 1.0	3.6 ± 1.3	12.4 ± 0.8	2.9 ± 0.4	9.6 ± 2.8	5.2 ± 0.9	19.3 ± 1.0
	Stomach	0.7 ± 0.1	1.9 ± 1.3	0.7 ± 0.1	2.6 ± 0.3	0.8 ± 0.2	4.2 ± 3.1	1.5 ± 0.3	2.4 ± 0.2
	Brain	0.2 ± 0.0	0.2 ± 0.1	0.2 ± 0.0	0.3 ± 0.0	0.3 ± 0.0	1.0 ± 0.1	0.7 ± 0.1	1.5 ± 0.1
	Blood	71.0 ± 4.3	31.7 ± 2.1	90.5 ± 5.9	72.5 ± 7.6	106.3 ± 5.2	71.0 ± 14.5	83.4 ± 7.7	49.7 ± 4.8
	Carcass	18.10 ± 2.32	31.35 ± 3.67	17.6 ± 1.5	21.2 ± 2.8	18.8 ± 1.6	22.4 ± 1.5	28.4 ± 2.0	26.1 ± 1.0
SUV	Kidneys	5.6 ± 0.6	1.6 ± 0.1	4.3 ± 0.7	2.5 ± 0.2	3.1 ± 0.6	2.5 ± 0.4	2.4 ± 0.3	2.4 ± 0.2
	Liver	3.2 ± 0.2	1.7 ± 0.3	1.5 ± 0.2	1.9 ± 0.3	1.4 ± 0.3	1.0 ± 0.1	1.5 ± 0.1	1.4 ± 0.2
	Spleen	1.6 ± 0.2	1.1 ± 0.3	0.9 ± 0.1	1.1 ± 0.2	2.1 ± 0.7	2.2 ± 0.8	1.9 ± 0.8	2.4 ± 0.4
	Lungs	3.5 ± 1.0	1.1 ± 0.4	6.0 ± 1.6	4.7 ± 0.7	9.3 ± 2.9	3.8 ± 0.4	6.2 ± 2.1	3.5 ± 1.1
	Heart	2.3 ± 1.3	0.5 ± 0.4	1.2 ± 0.2	1.0 ± 0.3	1.6 ± 0.4	1.7 ± 0.5	1.6 ± 0.4	0.9 ± 0.1
	Brain	0.2 ± 0.0	0.1 ± 0.0	0.2 ± 0.0	0.2 ± 0.0	0.5 ± 0.1	1.4 ± 0.2	0.7 ± 0.1	1.1 ± 0.1
	Blood	10.1 ± 0.6	4.5 ± 0.3	12.9 ± 0.8	10.4 ± 1.1	17.3 ± 2.5	11.1 ± 1.2	11.9 ± 1.1	7.1 ± 0.7

This activity is at 2 and 60 min p.i. Data are expressed as mean ± standard error (*n *= 3).

#### Small animal imaging studies

**Figure 4:**
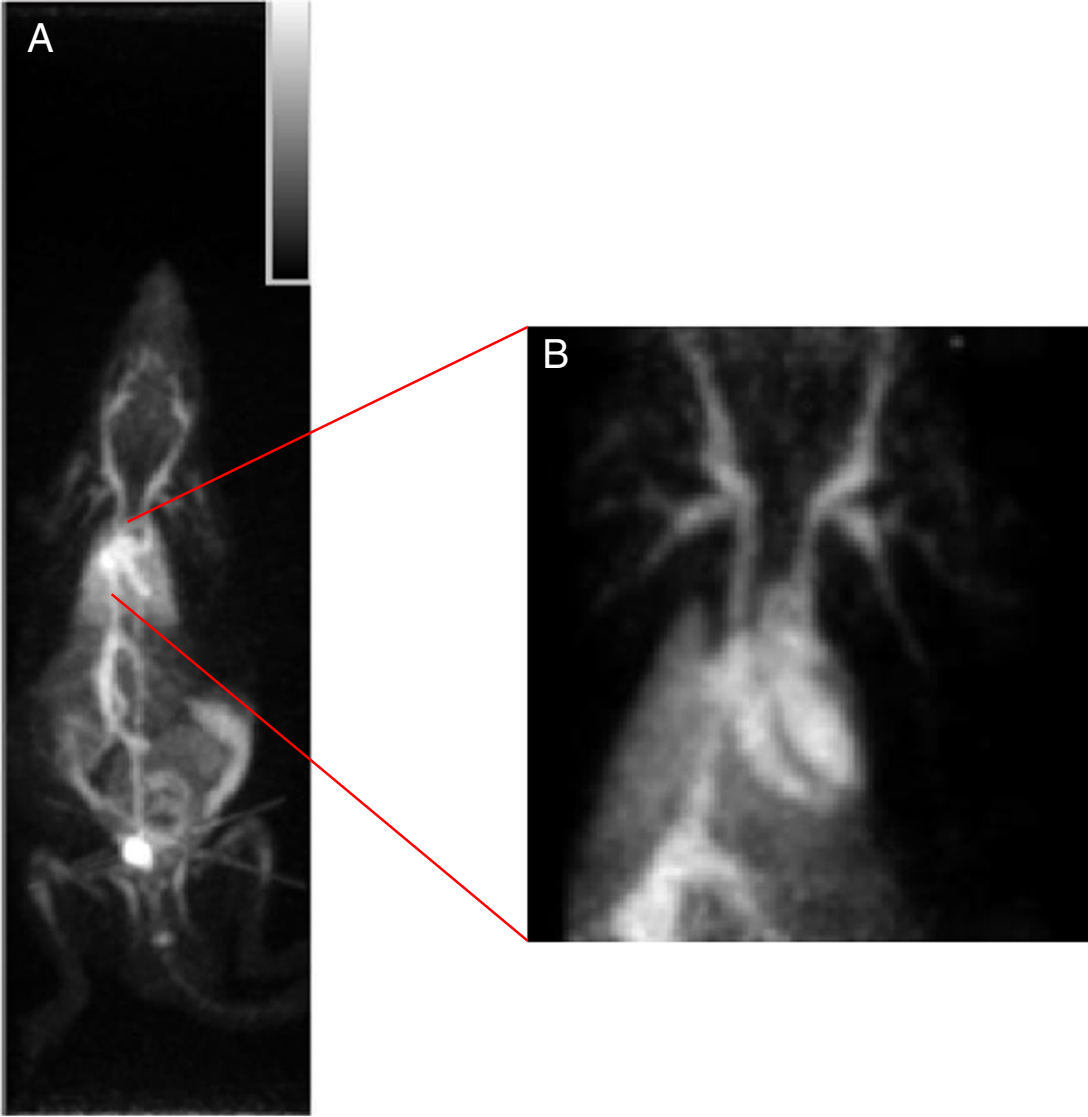
**Maximum intensity projection images. **(**A**) Maximum intensity projection micro-PET image after administration of 37 MBq of [^18^F]-(**5**), showing high activity in the heart and large blood vessels. (**B**) Maximum intensity projection detail of the heart showing high activity in the left and right ventricles of the heart and in all of the major blood vessels.

In view of the biodistribution results and the longer half-life of fluorine-18 (110 min) compared with carbon-11 (20 min), further imaging studies were performed with [^18^F]**-**(**5**). A proof-of-concept *in vivo* visualization of blood pool activity was carried out by acquiring a 1-h dynamic micro-PET scan in rats. The PET data revealed a high blood pool activity in the heart and excellent visualization of all major blood vessels with minimal leakage of tracer to the extravascular compartment (Figure 4).

**Figure 5:**
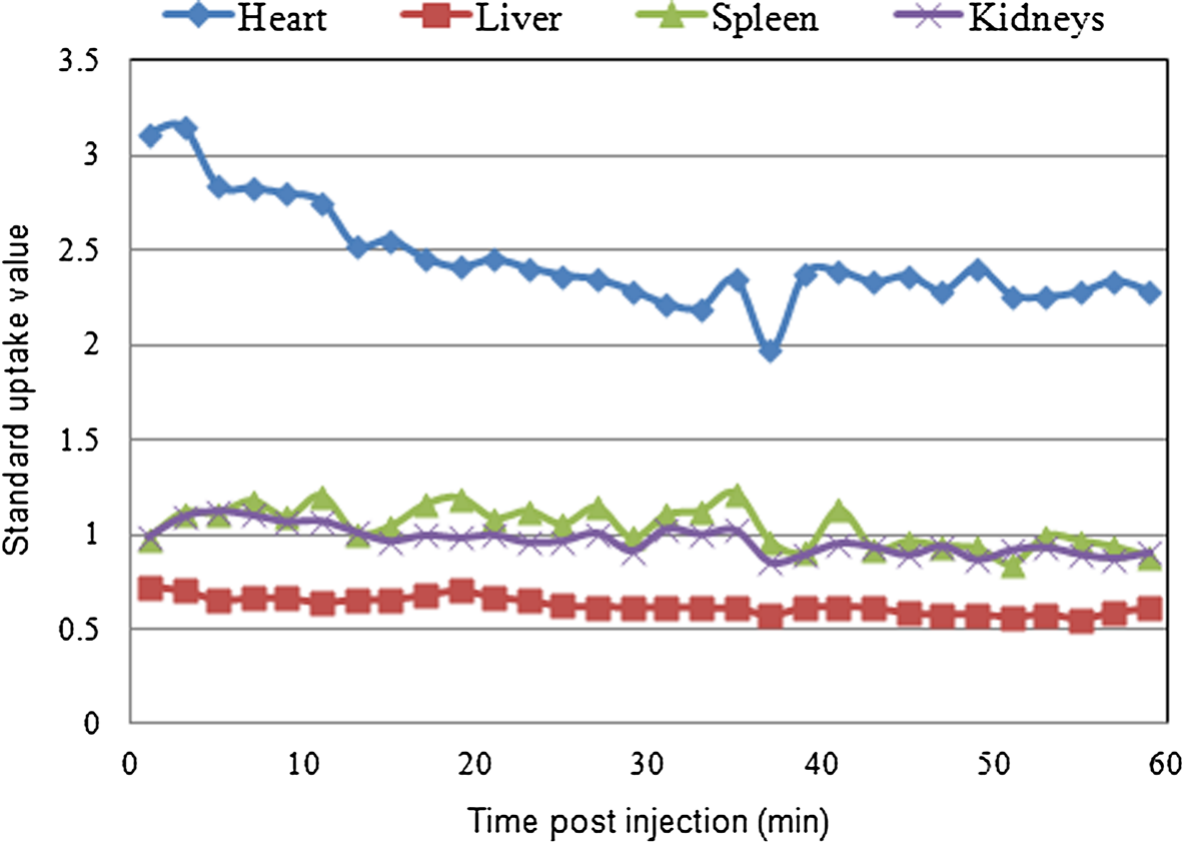
**Time activity curves of [**^**18**^**F]-(5) in various organs versus myocardial blood pool activity. **Time activity curves of [^18^F]-(**5**) in the liver, spleen, and kidneys of a Wistar rat in comparison with the myocardial blood pool activity illustrating good retention of tracer in the blood pool compartment with excellent target-to-background ratios.

**Figure 6:**
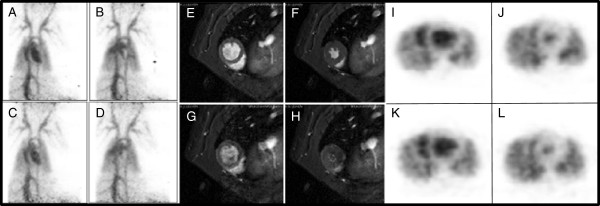
**Retrospective ECG-gated micro-PET and cMRI images of a representative rat. **(**A, E, I**) End-diastolic and (**B, F, J**) end-systolic frames during rest on micro-PET and cMRI, respectively. (**C, G, K**) End-diastolic and (**D, H, L**) end-systolic images during pharmacological stress in the same rat. The lower ESV in stress condition is clearly observed on both micro-PET and cMRI. (left panel: PET maximum intensity projection images, central panel: axial MRI images, right panel: axial PET images).

The time activity curves in rats (Figure 5) show that large blood pool organs such as the liver, spleen, and kidneys had a lower tracer uptake and retention compared with the heart (SUV of 2.5). The average SUV ratios of heart-to-spleen, heart-to-liver, and heart-to-kidney were found to be 2.3, 4, and 2.5 respectively, with an excellent target-to-background activity ratio. The LVEF values with micro-PET for rats 1 and 2 were found to be 75% and 61% at rest and under pharmacological stress 84% and 85%, respectively. Similarly, using cMRI, the LVEF values were found to be 53% and 73% at rest and under pharmacological stress 81% and 82%, respectively (Figure 6).

#### Pig study

**Figure 7:**
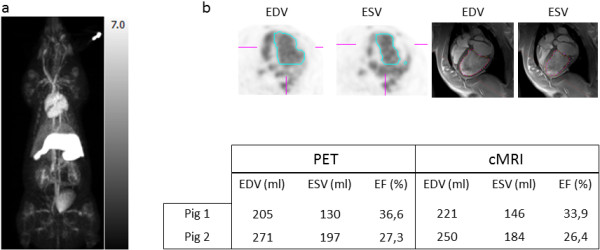
**Coronal maximum intensity projection of the pig model and quantitative evaluation with PET and MRI. **(**a**) Coronal maximum intensity projection of the pig model reveals high activity in the left and right atria and ventricles, in the spleen, and in all major blood vessels. (**b**) Representative image illustrating delineation of ESV and EDV on PET and cMRI. A good agreement was observed between PET and MRI for quantification of LV volumes and EF.

Whole body imaging obtained 60 min after tracer injection reveals high activity in the left and right atria and ventricles, in the spleen, and in all major blood vessels (Figure 7a).

Similar to small animal studies, quantitative evaluation of LV volumes and EF with PET shows a good agreement with values obtained by cMRI (Figure 7b).

## Discussion

The aim of this study was to develop PET tracers for *in vivo* labeling of RBCs and to evaluate their potential application for assessing cardiac function. CA isozyme II, an efficient catalyst involved in the physiological reaction of reversible hydration of carbon dioxide to bicarbonate and a proton, is abundantly present in the RBCs (17 to 20 μM) and can be functionally inhibited by sulfonamide derivatives [23]. Several highly specific and potent inhibitors targeting CA II were reported in the literature [24]. Here, we synthesized ^11^C and ^18^F sulfonamide derivatives in a straightforward reaction with good radiochemical yield and purity. To penetrate the cellular membrane and to interact with CA II localized in the cytosol, the tracer agents need to be lipophilic and have a PSA < 90 Å^2^[25,26]. The calculated PSA was found to be 69 Å^2^ for ^18^F]**-**(**5**) and ^11^C]**-**(**6**). Due to the additional amide bond, the cPSA value increased to 99 Å^2^ for ^18^F]**-**(**3**) and ^11^C]**-**(**4**). In order to be efficient tracers for *in vivo* labeling of erythrocytes, it is critical that the tracer agents rapidly diffuse in the RBC where they bind to CA I/II isozymes after intravenous injection. The fraction of the tracer which is not bound to the RBCs should be minimal at the time that the injection bolus reaches organs such as the liver or kidneys to avoid clearance through these excretory organs. It is thus anticipated that the labeling efficiency will be higher in larger species as not only a larger excess of CA I/II is available, but also the relative cardiac output is lower and the circulation time is slower. This is in accordance with the much slower blood clearance in man versus rats for the radioiodinate sulfonamides reported by Singh et al. [14].

The data of Table 1 show that the investigated compounds demonstrated lower inhibition constants for CA II and higher CA II vs. CA I specificity of compounds (**3**) and (**4**) versus compounds (**5**) and (**6**). Human whole blood analysis indicated that the tracer agents were retained ≥90% in RBCs at 10 and 60 min post incubation at room temperature, suggesting their membrane-permeable nature. Further experiments showed that more than 90% of the activity is confined to the RBCs and not to other constituents of the blood. In addition, the competition study with CA inhibitor AAZ indicates that the binding to RBCs is due to specific interaction with CA I/II. Biodistribution studies in mice showed significant accumulation of all tested tracer agents in the blood pool at 2 min with a relative decrease of 23% to 53% of the blood pool-associated activity at 60 minutes p.i. Compound [^18^F]-(**3**) shows only 32% ID at 60 min p.i. in the blood in contrast with its potent inhibitory activity against CA II (*K*_i_ = 8 nM) and its PSA value which is comparable to that of [^11^C]-(**4**) for which the blood retention at 60 min p.i. was twice as high (68%). The higher urinary excretion of ^18^F-labeled compounds compared to their carbon-11 analogs may be caused by urinary excretion of polar radiometabolites which may not be generated in the case of the ^11^C-labeled compounds.

The potential application to calculate LVEF values in rats and pigs was demonstrated in a proof-of-concept study with [^18^F]**-**(**5**). As shown in Figure 5, the highest SUV in rats was observed over the heart region for which the SUV value at 60 min amounted to 75% of the 2-min value. Furthermore, it is noteworthy that no bone uptake was observed, indicating the absence of *in vivo* defluorination. The presence of free fluoride would have an adverse effect with high background activity in the upper skeleton and would result in inaccurate quantification due to partial volume effects. The potential application of the tracer for assessing cardiac function was demonstrated by measuring the global parameters (LV volumes and EF) in rest and stress conditions using ECG micro-PET and cMRI. In stress condition, there was a good correlation of LVEF values between PET and cMRI. In rest condition, the obtained LVEF values were somewhat more variable for both techniques. This discrepancy can be attributed to subtle changes in the physiology of the animal even though every effort was made to obtain comparable study conditions, e.g., using a common anesthesia protocol.

In the pig model of myocardial infarction, an excellent agreement was found between the EF and LV values obtained with MRI and PET, respectively. The whole body image (Figure 7a) largely reflects blood pool with high activity in the heart and major blood vessels. In contrast to the human spleen, the pig's spleen stores about 20% of its total number of RBCs which explains the high activity observed in this organ [27]. A small amount of activity was also observed in the bladder.

In view of the low extravascular activity in the abdomen, this tracer can potentially be used for PET visualization of gastrointestinal bleeding and hemangioma with higher resolution and sensitivity compared with SPECT using technetium-labeled erythrocytes. Labeling of the RBCs with [^18^F]**-**(**5**) has the advantage that it can potentially be used for any species, and in addition, it does not require any manipulation of blood.

Both PET and cMRI endure certain limitations, especially in terms of manual delineation of ESV and EDV, which is subjected to a risk of partial volume effect on PET, and for cMRI, there is a risk for misdelineation of contours at the myocardium. Nevertheless, the study demonstrated the ability of the synthesized PET tracers to allow assessment of LVEF and volumes in rats and pigs. As mentioned above, a slower clearance from the blood pool compartment of these tracers in humans compared with rats and pigs is anticipated so that further clinical evaluation of this compound is warranted.

## Conclusions

Our results clearly demonstrate that the developed ^11^C- or ^18^F-labeled sulfonamide derivatives can be used for blood pool imaging using PET. Our initial *in vitro* and *in vivo* evaluation indicates the tracer's ability to efficiently radiolabel RBCs *in vivo*. Biodistribution and imaging studies revealed mainly blood pool activity with minimal background signal. In a proof-of-concept study, we demonstrated the applicability of ECG-gated PET to assess cardiac function and volumes in rats and in a pig model of myocardial infarction. Further studies are warranted to fully exploit the potential of this tracer and translate it to clinical applications.

## Competing interests

The authors declare that they have no competing interests.

## Authors’ contributions

Each author has contributed significantly to the submitted work: OG designed the study, carried out the PET imaging studies, analyzed the data, and wrote the manuscript. VA, SC, BJC, and RC carried out the (radio)synthesis, *in vitro* evaluation, and biodistribution studies, and VA co-wrote the work. TD and UH carried out and analyzed the micro-MRI studies, MK carried out the PET experiments, DD carried out the pig studies, and PC carried out and analyzed the pig MRI studies. JN, AMV, SJ, and GMB were responsible for the design and critical revision of the manuscript. All authors read and approved the final manuscript.

## Supplementary Material

Additional file 1 **Reference analogs (1–5) and production of the secondary radiolabeling agents**^**11**^**CH**_**3**_**I and**^**18**^**FEtBr. ** Click here for file
